# Machine Learning for Lung Cancer Diagnosis, Treatment, and Prognosis

**DOI:** 10.1016/j.gpb.2022.11.003

**Published:** 2022-12-01

**Authors:** Yawei Li, Xin Wu, Ping Yang, Guoqian Jiang, Yuan Luo

**Affiliations:** 1 Department of Preventive Medicine, Feinberg School of Medicine, Northwestern University, Chicago, IL 60611, USA; 2 Department of Medicine, University of Illinois at Chicago, Chicago, IL 60612, USA; 3 Department of Quantitative Health Sciences, Mayo Clinic, Rochester, MN 55905 / Scottsdale, AZ 85259, USA; 4 Department of Artificial Intelligence and Informatics, Mayo Clinic, Rochester, MN 55905, USA

**Keywords:** Omics dataset, Imaging dataset, Feature extraction, Prediction, Immunotherapy

## Abstract

The recent development of imaging and sequencing technologies enables systematic advances in the clinical study of lung cancer. Meanwhile, the human mind is limited in effectively handling and fully utilizing the accumulation of such enormous amounts of data. Machine learning-based approaches play a critical role in integrating and analyzing these large and complex datasets, which have extensively characterized lung cancer through the use of different perspectives from these accrued data. In this review, we provide an overview of machine learning-based approaches that strengthen the varying aspects of lung cancer diagnosis and therapy, including early detection, auxiliary diagnosis, prognosis **prediction**, and **immunotherapy** practice. Moreover, we highlight the challenges and opportunities for future applications of machine learning in lung cancer.

## Introduction

Lung cancer is one of the most frequently diagnosed cancers and the leading cause of cancer deaths worldwide. About 2.20 million new patients are diagnosed with lung cancer each year [1], and 75% of them die within five years of diagnosis [2]. High intra-tumor heterogeneity (ITH) and complexity of cancer cells giving rise to drug resistance make cancer treatment more challenging [3]. Over the past decades, the continuous evolution of technologies in cancer research has contributed to many large collaborative cancer projects, which have generated numerous clinical, medical imaging, and sequencing databases [4], [5], [6]. These databases facilitate researchers in investigating comprehensive patterns of lung cancer from diagnosis, treatment, and responses to clinical outcomes [7]. In particular, current studies on -omics analysis, such as genomics, transcriptomics, proteomics, and metabolomics, have expanded our tools and capabilities for research. Cancer studies are undergoing a shift toward the integration of multiple data types and mega sizes. However, using diverse and high-dimensional data types for clinical tasks requires significant time and expertise even with assistance from dimension reduction methods such as matrix and tensor factorizations [8], [9], [10], [11], and analyzing the exponentially growing cancer-associated databases poses a major challenge to researchers. Therefore, using machine learning (ML) models to automatically learn the internal characteristics of different data types to assist physicians’ decision-making has become increasingly important.

**Figure 1:**
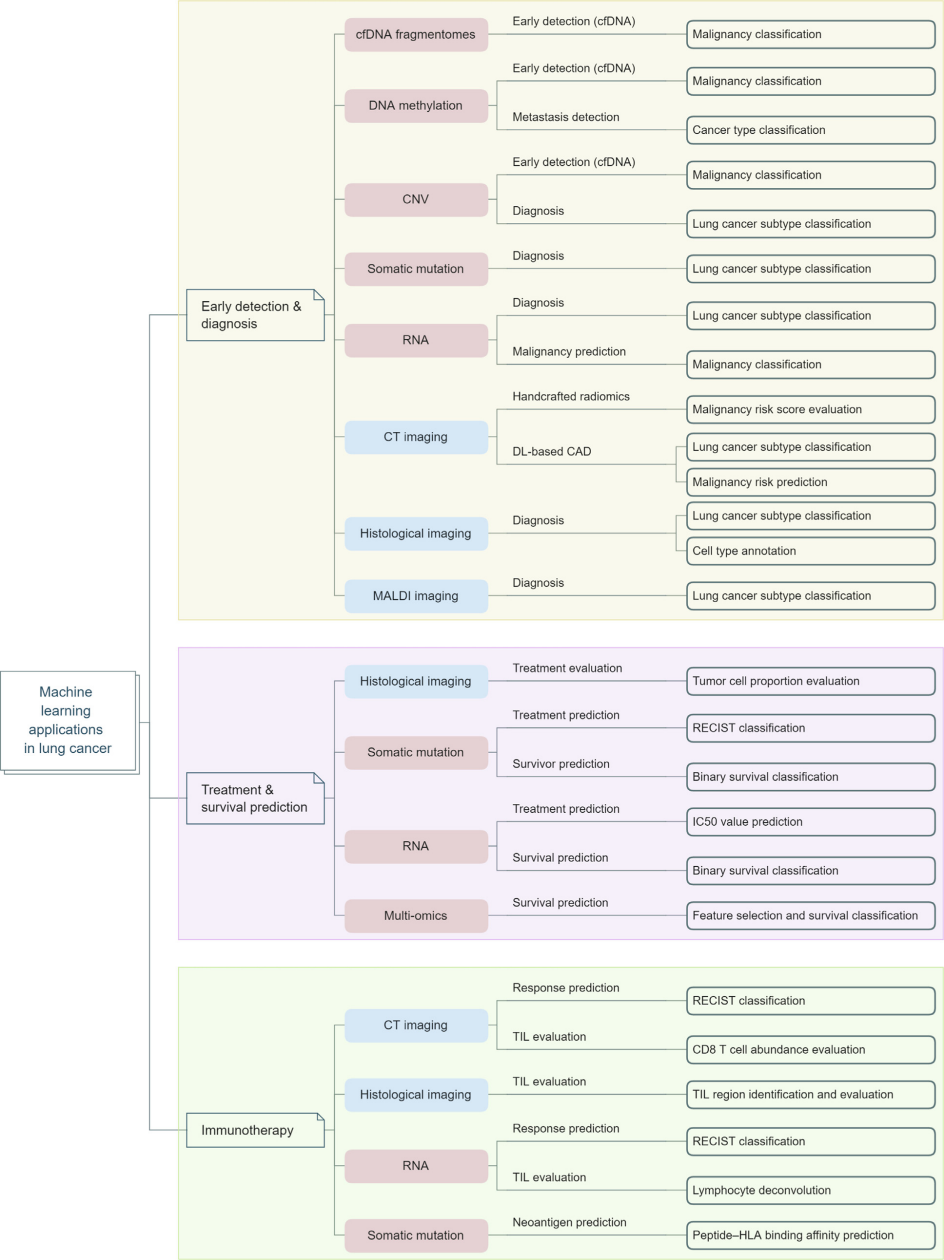
**Applications of ML model in lung cancer** We presented an overview of ML methodologies for different aspects of lung cancer therapies, including CAD from imaging datasets, lung cancer early detection based on sequencing technologies, data integration and biomarker extraction from multi-omics datasets, treatment response and prognosis prediction, and immunotherapy studies. ML, machine learning; IC50, half-maximal inhibitory concentration; HLA, human leukocyte antigen; CT, computed tomography; MALDI, matrix-assisted laser desorption/ionization; DL, deep learning; cfDNA, cell-free DNA; CAD, computer-aided diagnosis; CNV, copy number variation; RECIST, Response Evaluation Criteria in Solid Tumors; TIL, tumor-infiltrating lymphocyte.

ML is a subgroup of artificial intelligence (AI) that focuses on making predictions by identifying patterns in data using mathematical algorithms [12]. It has served as an assisting tool in cancer phenotyping and therapy for decades [13], [14], [15], [16], [17], [18], [19], and has been widely implemented in advanced approaches for early detection, cancer type classification, signature extraction, tumor microenvironment (TME) deconvolution, prognosis prediction, and drug response evaluation [20], [21], [22], [23], [24], [25], [26], [27]. Herein, we present an overview of the main ML algorithms that have been used to integrate complex biomedical data (*e.g.*, imaging or sequencing data) for different aspects of lung cancer (Figure 1; Tables S1 and S2), and outline major challenges and opportunities for future applications of ML in lung cancer clinical research and practice. We hope that this review promotes a better understanding of the roles and potentialities of ML in this field.

## Apply ML for early detection and auxiliary diagnosis of lung cancer

### ML on early detection and diagnosis using medical imaging datasets

**Figure 2:**
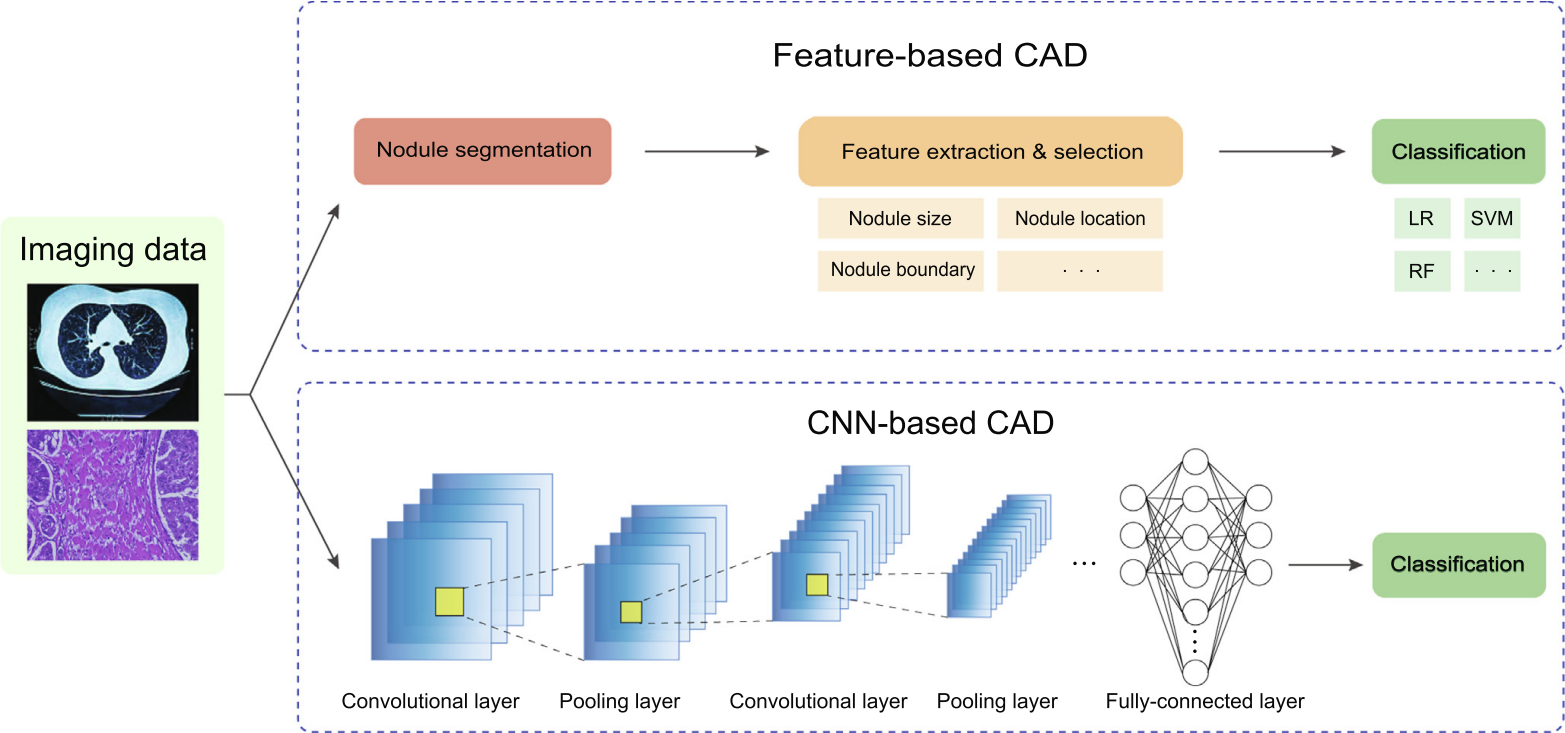
**Feature-based CAD and DL-based CAD systems** Differences in the development process of feature-based CAD systems and CNN-based CAD systems. Compared with feature-based CAD systems, the DL-based CAD systems can automatically retrieve and extract intrinsic features of a suspicious nodule. CNN, convolutional neural network; LR, logistic regression; SVM, support vector machine; RF, random forest.

Early diagnosis is an important procedure for reducing deaths related to lung cancer. Chest screening using low-dose computed tomography (CT) is the primary approach for the surveillance of people with increased lung cancer risk. To promote diagnostic efficiency, the computer-aided diagnosis (CAD) system was developed to assist physicians in the interpretation of medical imaging data [28], [29], which has been demonstrated as a useful second opinion for physicians [30]. The traditional feature-based CAD task can be broken into three steps: nodule segmentation, feature extraction and selection, and clinical judgment inference (classification) (Figure 2). Some approaches apply the measured texture features of specified nodules in CT images combined with the patient’s clinical variables as input features to train an ML classifier, including logistic regression (LR) [31], [32], [33] or linear discriminant analysis (LDA) [34], for malignancy risk estimation. Typically, these measurements include nodule size, nodule type, nodule location, nodule count, nodule boundary, and emphysema information in CT images, and the clinical variables include the patient’s age, gender, specimen collection timing, family history of lung cancer, smoking exposure, and more. However, these features are mostly subjective and arbitrarily defined, and usually fail to achieve a complete and quantitative description of malignant nodule appearances.

With the development of deep learning (DL) algorithms, especially convolutional neural networks (CNNs), more studies have been conducted to apply DL-based models in the CAD system to improve its accuracy and reduce its false positive rate and execution time during lung tumor detection (Table 1) [35], [36]. Similar to feature-based CAD system, the workflow of these models usually consists of three steps: nodule detection and segmentation, nodule feature extraction, and clinical judgment inference [37]. Compared with traditional feature-based CAD systems, the DL-based CAD system can automatically retrieve and extract intrinsic features of a suspicious nodule [38], [39], and can model the 3D shape of a nodule (Figure 2). For example, Ciompi et al. [40] designed a model based on OverFeat [41], [42] by extracting three 2D-view-feature vectors (axial, coronal, and sagittal) of the nodule from CT scans. The recently integrated CNN models facilitate a global and comprehensive inspection of nodules for feature characterization from CT images. Buty et al. [37] designed a complementary CNN model, where a spherical harmonic model [43] for nodule segmentation was used to obtain the shape descriptions (“shape” feature) of the segmented nodule and a deep convolutional neural network (DCNN)-based model [41] to extract the texture and intensity features (“appearance” feature) of the nodule. The downstream classification relied on the combination of “shape” and “appearance” features. Similarly, Venkadesh et al. [44] used an ensemble model from two different models, 2D-ResNet50-based [45] and 3D-Inception-V1 [46], to respectively extract two features of a pulmonary nodule, and then concatenated the two features as the input features for classification. A superiority of the ensemble CNN model is that it can accurately identify malignant nodules from different sizes of nodules using the raw CT images. Benefiting from the features extracted from state-of-the-art CNN models, clinical judgment inference can be implemented through frequent ML techniques, including LR, random forest (RF), support vector machine (SVM), and neural networks (NNs). Notably, some studies also employed CNN models for final clinical judgment inference. Ardila et al. [47] proposed an end-to-end approach to systematically model both localization and lung cancer risk categorization tasks using the input CT data alone. Their approach was based on a combination of three CNN models: a Mask-RCNN [48] model for lung tissue segmentation, a modified RetinaNet [49] model for cancer region of interest (ROI) detection, and a full-volume model based on 3D-inflated Inception-V1 [50], [51] for malignancy risk prediction. In addition to CT images, CNN-based models are also widely used in histological imaging to help with lung cancer diagnosis. Compared with CT imaging, histological imaging can provide more biological information about cancer at the cellular level. To this end, AbdulJabbar et al. [52] used the Micro-Net [53] model to identify tissue boundaries followed by an SC-CNN [54] model to segment individual cells from hematoxylin and eosin (H&E)-stained and immunohistochemistry (IHC) images. The segmented cells were then applied for cell type classification to evaluate the proportions of each cell type in the images. This model helps to identify the differential evolution and immune evasion mechanisms between lung adenocarcinoma (LUAD) and lung squamous cell carcinoma (LUSC) with high resolution. Another study [55] utilized the Inception-V3 network [51] to classify whether the tissue was LUAD, LUSC, or normal from H&E-stained histopathology whole-slide images. A highlight of this study is that the model can also predict whether a given tissue has somatic mutations in several lung cancer driver genes, including *STK11*, *EGFR*, *FAT1*, *SETBP1*, *KRAS*, and *TP53*. Note that considering the high complexity and large resources of the datasets, some studies utilized transfer learning to improve their efficiency and robustness when training new models [38], [55].

**Table 1:** **Publications relevant toMLon early detection and diagnosis using imaging data**

**Publication**	**Feature extraction**	**Classification model**	**Sample size**	**Imaging data type**	**Performance**	**Validation method**	**Feature selection/input**	**Highlight/**a**dvantage**	**Shortcoming**
McWilliams et al. [31]	NA	LR	2961	CT images	AUC (0.907–0.960)	Hold-out	Clinical risk factors + nodule characteristics on CT images	Using the extracted feature as input, the classifier can achieve high AUC in small nodules (< 10 mm)	The selection of nodule characteristics affects the predictive performance of the model
Riel et al. [32]	NA	LR	300	CT images	AUC (0.706–0.932)	Hold-out	Clinical factors + nodule characteristics on CT images	The classifier can perform equivalently as human observers for malignant and benign classification	The performance heavily relies on nodule size as the discriminator, and is not robust in small nodules
Kriegsmann et al. [34]	NA	LDA	326	MALDI	Accuracy (0.991)	Hold-out	Mass spectra from ROIs of MALDI image	The model maintains high accuracy on FFPE biopsies	The performance relies on the quality of the MALDI stratification
Buty et al. [37]	Spherical harmonics [44]; DCNN [41]	RF	1018	CT images	Accuracy (0.793–0.824)	10-fold cross-validation	CT imaging patches + radiologists’ binary nodule segmentations	The model reaches higher predictive accuracy by integrating shape and appearance nodule imaging features	No benchmarking comparisons were used in the study
Hussein et al. [38]	3D CNN-based multi-task model	3D CNN-based multi-task model	1018	CT images	Accuracy (0.9126)	10-fold cross-validation	3D CT volume feature	The model achieves higher accuracy than other benchmarked models	The ground truth scores defined by radiologists for the benchmark might be arbitrary
Khosravan et al. [39]	3D CNN-based multi-task model	3D CNN-based multi-task model	6960	CT images	Segmentation DSC (0.91); classification accuracy (0.97)	10-fold cross-validation	3D CT volume feature	The model integration of clustering and sparsification algorithms helps to accurately extract potential attentional regions	Segmentation might fail if the ROIs are outside the lung regions
Ciompi et al. [40]	OverFeat [42]	SVM; RF	1729	CT images	AUC (0.868)	10-fold cross-validation	3D CT volume feature, nodule position coordinate, and maximum diameter	This is the first study attempting to classify whether the diagnosed nodule is benign or malignant	The model requires specifying the position and diameter of the nodule as input, but many nodules could not be located on the CT images
Venkadesh et al. [44]	2D-ResNet50-based [45]; 3D-Inception-V1 [46]	An ensemble model based on two CNN models	16,429	CT images	AUC (0.86–0.96)	10-fold cross-validation	3D CT volume feature and nodule coordinates	The model achieves higher AUC than other benchmarked models	The model requires specifying the position of the nodule, but many nodules are unable to be located on the CT images
Ardila et al. [47]	Mask-RCNN [48]; RetinaNet [49]; 3D-inflated Inception-V1 [50], [51]	Mask-RCNN [48]; RetinaNet [49]; 3D-inflated Inception-V1 [50], [51]	14,851	CT images	AUC (0.944)	Hold-out	Patient’s current and prior (if available) 3D CT volume features	The model achieves higher AUC than radiologists when samples do not have prior CT images	The training cohort is from only one dataset, although the sample size is large
AbdulJabbar et al. [52]	Micro-Net [53]; SC-CNN [54]	An ensemble model based on SC-CNN [54]	100	Histological images	Accuracy (0.913)	Hold-out	Image features of H&E-stained tumor section histological slides	The model can annotate cell types at the single-cell level using histological images only	The annotation accuracy is affected by the used reference dataset
Coudray et al. [55]	Multi-task CNN model based on Inception-V3 [51]	Multi-task CNN model based on Inception-V3 network [51]	1634	Histological images	AUC (0.733–0.856)	Hold-out	Transformed 512 × 512-pixel tiles from nonoverlapping ‘patches’ of the whole-slide images	The model can predict whether a given tissue has somatic mutations in genes *STK11*, *EGFR*, *FAT1*, *SETBP1*, *KRAS*, and *TP53*	The accuracy of the gene mutation prediction is not very high
Lin et al. [59]	DCGAN [58] + AlexNet [41]	DCGAN [58] + AlexNet [41]	22,489	CT images	Accuracy (0.9986)	Hold-out	Initial + synthetic CT images	The model uses GAN to generate synthetic lung cancer images to reduce overfitting	No benchmarking comparisons were used
Ren et al. [60]	DCGAN [58] + VGG-DF	DCGAN [58] + VGG-DF	15,000	Histopathological images	Accuracy (0.9984); F1-score (99.84%)	Hold-out	Initial + synthetic histopathological images	The model uses GAN to generate synthetic lung cancer images and a regularization-enhanced model to reduce overfitting	The dimension of images by generator (64 × 64) is not sufficient for biomedical domain

*Note*: ML, machine learning; NA, not applicable; LR, logistic regression; AUC, area under the curve; CT, computed tomography; LDA, linear discriminant analysis; MALDI, matrix-assisted laser desorption/ionization; ROI, region of interest; FFPE, formalin-fixed paraffin-embedded; CNN, convolutional neural network; DSC, dice similarity coefficient; SVM, support vector machine; RF, random forest; DCNN, deep convolutional neural network; SC-CNN, spatially constrained convolutional neural network; DCGAN, deep convolutional generative adversarial network; RCNN, Region-CNN; H&E, hematoxylin and eosin; 2D, two dimensional; 3D, three dimensional. Compared with hold-out, cross-validation is usually more robust, and accounts for more variance between possible splits in training, validation, and test data. However, cross-validation is more time consuming than using the simple holdout method.

Though these ML algorithms are already widely used in CAD, the challenge is that only a limited number of the images are labeled. Training a complex CNN model using a limited number of training sets may result in overfitting. Recently, generative adversarial network (GAN)-based models have been used to improve the performance of discriminative classifiers by generating pseudo images [56]. Chuquicusma et al. [57] first employed a deep convolutional GAN (DCGAN) [58] model to generate synthetic lung nodule CT scans. With their work, more recent studies have integrated the GAN models with other CNN models to address the overfitting problem in lung cancer classification. Lin et al. [59] used a two-step model — a DCGAN to generate synthetic lung cancer images and an AlexNet [41] for lung cancer classification using both original and synthetic datasets. Similar work was also done by Ren and colleagues [60]. They also used DCGAN [58] for data augmentation. To improve performance, they then designed a regularization-enhanced transfer learning model called VGG-DF for data discrimination to prevent overfitting problems with pre-trained model auto-selection.

### ML on early detection and diagnosis using -omics sequencing datasets

**Figure 3:**
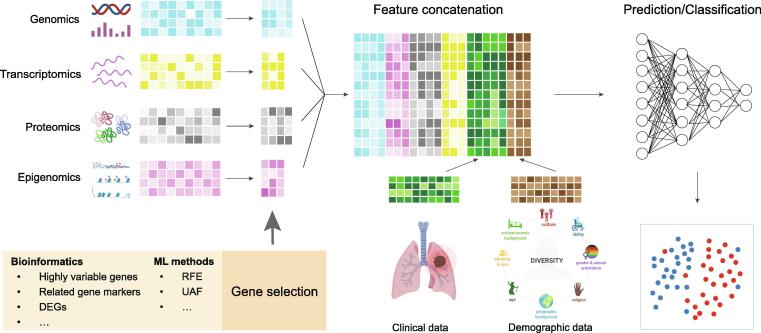
**Omics analysis in lung cancer studies** Different sequencing techniques allow for the simultaneous measurement of multiple molecular features of a biological sample. To improve efficiency and reduce overfitting, statistical and ML tools perform differential analysis or feature selection. Further ML models concatenate the obtained omics features with clinical features as input for lung cancer diagnostic/prognostic prediction. DEG, differentially expressed gene; RFE, recursive feature elimination; UAF, univariate association filtering.

Although periodic medical imaging tests are recommended for high-risk populations, implementation has been complicated by a high false discovery rate [61], [62]. Therefore, there is a critical need for new techniques in early detection of lung cancers. Recent sequencing technologies enable diverse methods for early detection of lung cancer [63]. In the meantime, accurately classifying lung cancer subtypes is crucial in guiding optimal therapeutic decision-making. LUAD (∼ 45%) and LUSC (∼ 25%) are the two most common subtypes of lung cancer but are often treated similarly except for targeted therapy [64]. However, studies have indicated that LUAD and LUSC have drastically different biological signatures, and they have suggested that LUAD and LUSC should be classified and treated as different cancers [65], [66]. From a computational perspective, both early detection and subtype identification are part of the classification task. Previous ML studies have shown the efficiency and advancement of early detection and cancer type classification in large pan-cancer sequencing datasets [67], [68], [69], [70], [71], [72], [73], [74], [75], which may provide evidence for lung cancer diagnosis. It is known that cancer cells are characterized by many genetic variations, and the accumulation of these genetic variations can be signatures that document the mutational patterns of different cancer types [3], [5], [76], [77]. For this reason, recent studies have concentrated on extracting better genomic signatures as input features to boost the accuracy of their ML models. For early detection, blood-based liquid biopsy, including cell-free DNA (cfDNA) fragments, circulating tumor DNA (ctDNA), microRNA (miRNA), methylation, exosomes, and circulating tumor cells (CTCs), to explore potential circulating tumor signatures is considered a reliable method [63] (Figure 3). Integrating these liquid biopsy signatures, many discriminative models (SVM, RF, and LR) have been used to detect tumors with high discovery rates [78], [79], [80], [81]. For lung cancer subtype classification, somatic mutations, including single-nucleotide variants (SNVs), insertions, and deletions, usually have specific cancer type profiles [82]. Thus, studies have leveraged somatic mutations as input features to train classifiers for LUAD–LUSC classification [83]. Many of these mutations, especially driver mutations, can change expression levels, which impact gene function and interrupt cellular signaling processes [82]. As a result, different cancer types show different expression levels of certain proteins [84], [85]. Imposed by these unique expression profiles of cancer type, ML models can leverage RNA sequencing as input data to categorize the malignancy (benign or malignant) and subtypes (LUAD or LUSC) of patients [86], [87], [88], [89]. Similarly, copy number variation (CNV) is reported to be highly correlated with differential gene expression [90], and can be ubiquitously detected in cancer cells. As such, CNVs can also be used to train ML models for cancer type classification in lung cancer studies [81], [91], [92]. Note that Daemen et al. [92] proposed a recurrent hidden Markov model (HMM) for the identification of extended chromosomal regions of altered copy numbers, which offers high accuracy for classification. More recently, Jurmeister et al. [93] used DNA methylation profiles as input features to determine if the detected malignant nodule is primary lung cancer or the metastasis of another cancer. Directly using all generated genes as an input feature may result in overfitting [94]. Thus, many studies used different computational approaches to select multiple cancer-associated genes to enhance their ML models (Figure 3). Some studies used ML-based algorithms for feature selection. For example, Liang et al. [80] and Whitney et al. [86] employed the least absolute shrinkage and selection operator (LASSO) method to select the optimal markers for model training; Aliferis et al. [89] utilized recursive feature elimination (RFE) [95] and univariate association filtering (UAF) models to select highly cancer-associated genes. In addition, using unsupervised models for sample population subtype clustering, and then identifying each cluster’s marker genes is also seen in many studies [96], [97]. Apart from ML-based models, some studies used statistical methods for feature selection. Raman et al. [81] designed a copy number profile abnormality (CPA) score to reinforce the CNV feature which is more robust and less subject to variable sample quality than directly using CNVs as the input feature. Daemen et al. [92] integrated several statistical tests (ordinary fold changes, ordinary *t*-statistics, SAM-statistics, and moderated *t*-statistics) to select a robust differential expression gene set. Aside from these single-measured signatures, some studies [81], [86], [88] combined the -omics signatures with clinical signatures to achieve better results. Using these tumor-type specific -omics signatures, many algorithms, K-nearest neighbors (KNN), naive Bayes (NB), SVM, decision tree (DT), LR, RF, LDA, gradient boosting, and NN, have demonstrated their ability to accurately detect and classify different lung cancer patterns (Table 2). Note that to improve the accuracy of ML models, Kobayashi et al. [83] added an element-wise input scaling for the NN model, which allows the model to maintain its accuracy with a small number of learnable parameters for optimization.

**Table 2:** **Publications relevant toMLon early detection and diagnosis using sequencing data**

**Publication**	**ML method**	**Sample size**	**Sequencing data type**	**Performance**	**Validation method**	**Feature selection**	**Highlight/**a**dvantage**	**Shortcoming**
Mathios et al. [78]	LR model with a LASSO penalty	799	cfDNA fragment	AUC (0.98)	10-fold cross-validation	cfDNA fragment features, clinical risk factors, and CT imaging features	This study provides a framework for combining cfDNA fragmentation profiles with other markers for lung cancer detection	DNA variations in late-stage disease may affect cfDNA detection
Lung-CLiP [79]	5-nearest neighbor; 3-nearest neighbor; NB; LR; DT	160	cfDNA	AUC (0.69–0.98)	Leave-one-out cross-validation	SNV + CNV features	This study establishes an ML framework for the early detection of lung cancers using cfDNA	Sampling bias exists (most are smokers) in the training dataset
Liang et al. [80]	LR	296	ctDNA	AUC (0.816)	10-fold cross-validation	Nine DNA methylation markers	This study establishes an ML framework for the early detection of lung cancers using DNA methylation markers	The selected features are comprised of only nine methylation biomarkers, which poses a limitation on assay performance
Raman et al. [81]	RF; SVM; LR with ridge, elastic net; LASSO regularization	843	cfDNA	mAUC (0.896–0.936)	Leave-one-out cross-validation	Copy number profiling of cfDNA	The model provides a framework for using copy number profiling of cfDNA as a biomarker in lung cancer detection	Feature selection methods can be used to reduce overfitting and may have the potential to achieve higher AUC
Kobayashi et al. [83]	Diet Networks with EIS	954	Somatic mutation	Accuracy (0.8)	5-fold cross-validation	SNVs, insertions, and deletions across 1796 genes	The EIS helps to stabilize the training process of Diet Networks	The interpretable hidden interpretations obtained from EIS may vary between different datasets
Whitney et al. [86]	LR	299	RNA-seq of BECs	AUC (0.81)	10-fold cross-validation	Lung cancer-associated and clinical covariate RNA markers	The model keeps sensitivity for small and peripheral suspected lesions	The selected genes vary greatly under different feature selection processes and parameters
Podolsky et al. [87]	KNN; NB normal distribution of attributes; NB distribution through histograms; SVM; C4.5 DT	529	RNA-seq	AUC (0.91)	Hold-out	RNA-seq	This study systematically compares different models of lung cancer subtype classification across different datasets	Feature selection methods can be used to reduce overfitting
Choi et al. [88]	An ensemble model based on elastic net LR; SVM; hierarchical LR	2285	RNA-seq of bronchial brushing samples	AUC (0.74)	5-fold cross-validation	RNA-seq of 1232 genes with clinical covariates	The model integrates RNA-seq features and clinical information to improve the accuracy of risk prediction	Sample sizes in certain subgroups are small and may cause unbalanced training
Aliferis et al. [89]	Linear SVM; polynomial-kernel SVM; KNN; NN	203	RNA-seq	AUC (0.8783–0.9980)	5-fold cross-validation	RNA-seq of selected genes using RFE and UAF	The study uses different gene selection algorithms to improve the classification accuracy	The selected genes vary greatly across different training cohorts
Aliferis et al. [91]	DT; KNN; linear SVM; polynomial-kernel SVM; RBF-kernel SVM; NN	37	CNV measured by CGH	Accuracy (0.892)	Leave-one-out cross-validation	Copy number of 80 selected genes based on linear SVM	The study systematically compares different models of lung cancer subtype classification	The sample size is small
Daemen et al. [92]	HMM; weighted LS-SVM	89	CNV measured by CGH	Accuracy (0.880–0.955)	10-fold cross-validation	CNV measured by CGH	The use of recurrent HMMs for CNV detection provides high accuracy for cancer classification	Benchmarked comparisons are needed to demonstrate the superiority of using the HMM model
Jurmeister et al. [93]	NN; SVM; RF	972	DNA methylation	Accuracy (0.878–0.964)	5-fold cross-validation	Top 2000 variable CpG sites	The study provides a framework for using DNA methylation data to predict tumor metastases	The model cannot accurately predict samples with low tumor cellularity through methylation data

*Note*: LASSO, least absolute shrinkage and selection operator; cfDNA, cell-free DNA; NB, naive Bayes; DT, decision tree; SNV, single-nucleotide variant; CNV, copy number variation; ctDNA, circulating tumor DNA; mAUC, mean area under the curve; EIS, element-wise input scaling; BEC, bronchial epithelial cell; KNN, K-nearest neighbors; NN, neural network; RFE, recursive feature elimination; UAF, univariate association filtering; CGH, comparative genomic hybridization; HMM, hidden Markov model; LS-SVM, least squares support vector machines; RNA-seq, RNA sequencing. Compared with hold-out, cross-validation is usually more robust, and accounts for more variance between possible splits in training, validation, and test data. However, cross-validation is more time consuming than using the simple holdout method.

## Apply ML to lung cancer treatment response and survival prediction

### Prognosis and therapy response prediction

**Figure 4:**
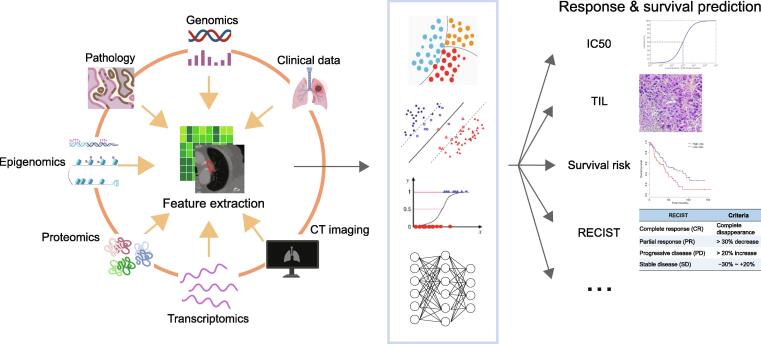
**Diagram of ML applications in treatment response and survival prediction**

Sophisticated ML models have acted as supplements for cancer intervention response evaluation and prediction [98], [99], and have demonstrated advances in optimizing therapy decisions that improve chances of successful recovery (Figure 4; Table 3) [100], [101]. There are several metrics that are available for evaluating cancer therapy response, including the Response Evaluation Criteria in Solid Tumors (RECIST) [102]. The definition of RECIST relies on imaging data, mainly CT and magnetic resonance imaging (MRI), to determine how tumors grow or shrink in patients [103]. To track the tumor volume changes from CT images, Jiang et al. [104] designed an integrated CNN model. Their CNN model used two deep networks based on a full-resolution residual network [105] model by adding multiple residual streams of varying resolutions, so that they could simultaneously combine features at different resolutions for segmenting lung tumors. Using the RECIST criterion, Qureshi [106] set up a RF model to predict the RECIST level under EGFR tyrosine kinase inhibitor (TKI) therapy given the patient’s mutation profile in gene *EGFR*. To improve the prediction performance, the model integrated clinical information, geometrical features, and energy features obtained from a patient’s *EGFR* mutant drug complex as input to train the classifiers. In a recent study, the authors defined a different metric, tumor proportional scoring (TPS) calculated as the percentage of tumor cells in digital pathology images, to evaluate the lung cancer treatment response [107]. They applied the Otsu threshold [108] with an auxiliary classifier generative adversarial network (AC-GAN) model to identify positive tumor cell regions (TC^+^) and negative tumor cell regions (TC^−^). And they ultimately used the ratio between the pixel count of the TC^+^ regions and the pixel count of all detected tumor cell regions to evaluate the TPS number. Another study from Geeleher et al. [109] used half-maximal inhibitory concentration (IC50) to evaluate drug response. In their model, the authors applied a ridge regression model [110] to estimate IC50 values for different cell lines in terms of their whole-genome expression level. More recently, Quiros et al. [111] established a phenotype representation learning (PRL) through self-supervised learning and community detection for spatial clustering cell type annotation on histopathological images. Their clustering results can be further used for tracking histological tumor growth patterns and identifying tumor recurrence. Indeed, their model has also demonstrated good performance in the LUAD and LUSC classifications.

**Table 3:** **Publications relevant toMLon treatment response and survival prediction**

**Publication**	**Feature extraction method**	**Prediction model**	**Sample size**	**Data type**	**Performance**	**Validation method**	**Feature selection/**i**nput**	**Highlight/**a**dvantage**	**Shortcoming**
Jiang et al. [104]	MRRN-based model	MRRN-based model	1210	CT Images	DSC (0.68–0.75)	5-fold cross-validation	3D image features	The model can accurately track the tumor volume changes from CT images across multiple image resolutions	The model does not predict accurately enough when the tumor size is small
Qureshi [106]	NA	RF; SVM; KNN; LDA; CART	201	Molecular structure and somatic mutations of EGFR	Accuracy (0.975)	10-fold cross-validation	4 clinical features + 4 protein drug interaction features + 5 geometrical features	The model integrates multiple features for data training, and achieves better performance than other benchmarked models	Among the possible 594 EGFR mutations available in the COSMIC database, the model only considers the most common 33 EGFR mutations for model training
Kapil et al. [107]	AC-GAN	AC-GAN	270	Digital pathology images	Lcc (0.94); Pcc (0.95); MAE (8.03)	Hold-out	PD-L1-stained tumor section histological slides	The model achieves better performance than other benchmarked, fully supervised models	In the experiments, the use of PD-L1 staining for TPS evaluation may not be accurate enough
Geeleher et al. [109]	NA	Ridge regression model	62	RNA-seq	Accuracy (0.89)	Leave-one-out cross-validation	Removed low variable genes	The model can accurately predict the drug response using RNA-seq profiles only	The training sample size is small
Chen et al. [123]	Chi-square test + NN	NN	440	RNA-seq	Accuracy (0.83)	Hold-out	RNA-seq of 5 genes	The model uses multiple laboratory datasets for training to improve its robustness	The model doesn’t consider demographic and clinical features, which may affect the prediction
LUADpp [125]	Top genes with most significant mutation frequency difference	SVM	371	Somatic mutations	Accuracy (0.81)	5-fold cross-validation	Somatic mutation features in 85 genes	The model can predict with high accuracy with only seven gene mutation features	Mutation frequency may be impacted by the sampling bias across datasets; LD may also affect the feature selection
Cho et al. [126]	Information gain; Chi-squared test; minimum redundancy maximum relevance; correlation algorithm	NB; KNN; SVM; DT	471	Somatic mutations	Accuracy (0.68–0.88)	5-fold cross-validation	Somatic mutation features composed of 19 genes	To improve performance, the model uses four different methods for feature selection	The training cohort consists of only one dataset
Yu et al. [128]	Information gain ratio; hierarchical clustering	RF	538	Multi-omics (histology, pathology reports, RNA, proteomics)	AUC (> 0.8)	leave-one-out cross-validation	15 gene set features	The study uses an integrative omics-pathology model to improve the accuracy in predicting patients’ prognosis	Cox models may be overfitted in multiple-dimension data
Asada et al. [130]	Autoencoder + Cox-PH + K-means + ANOVA	SVM	364	Multi-omics (miRNA, mRNA)	Accuracy (0.81)	Hold-out	20 miRNAs + 25 mRNAs	The study uses ML algorithms to systematically model feature extraction from multi-omics datasets	The model does not consider the impact of clinical and demographic variances in data training
Takahashi et al. [131]	Autoencoder + Cox-PH + K-means + XGBoost/LightGBM	LR	483	Multi-omics (mRNA, somatic mutation, CNV, mythelation, RPPA)	AUC (0.43–0.99 under different omics data)	Hold-out	12 mRNAs, 3 miRNAs, 3 methylations, 5 CNVs, 3 somatic mutations, and 3 RPPA	The study uses ML algorithms to systematically model feature extraction from multi-omics datasets	The datasets collected in this study contain uncommon samples between different omics datasets, which may cause bias in model evaluation
Wiesweg et al. [136]	Lasso regression	SVM	122	RNA-seq	Significant hazard ratio differences	Hold-out	7 genes from feature selection model + 25 cell type-specific genes	The ML-based feature extraction model performs better than using any single immune marker for immunotherapy response prediction	The metrics used in this study does not perceptual intuition. Using accuracy or AUC may be better
Trebeschi et al. [137]	LR; RF	LR; RF	262	CT imaging	AUC (0.76–0.83)	Hold-out	10 radiographic features	The model can extract potential predictive CT-derived radiomic biomarkers to improve immunotherapy response prediction	The predictive performance between different cancer types is not robust
Saltz et al. [142]	CAE [143]	VGG16 [144] + DeconvNet [145]	4612 (13 cancer types)	Histological images	AUC (0.9544)	Hold-out	Image features of H&E-stained tumor section histological slides	The model outperforms pathologists and other benchmarked models	The predictive performance between different cancer types is not robust

*Note*: MRRN, resolution residually connected network; CART, classification and regression trees; AC-GAN, auxiliary classifier generative adversarial networks; Lcc, Lin’s concordance coefficient; Pcc, Pearson correlation coefficient; MAE, mean absolute error; TPS, tumor proportional scoring; LD, linkage disequilibrium; Cox-PH, Cox proportional-hazards; ANOVA, analysis of variance; miRNA, microRNA; RPPA, reverse phase protein array; CAE, convolutional autoencoder; mRNA, messenger RNA; PD-L1, programmed cell death 1 ligand 1; COSMIC, the Catalogue Of Somatic Mutations In Cancer; EGFR, epidermal growth factor receptor. Compared with hold-out, cross-validation is usually more robust, and accounts for more variance between possible splits in training, validation, and test data. However, cross-validation is more time consuming than using the simple holdout method.

### Survival prediction

Prognosis and survival prediction as a part of clinical oncology is a tough but essential task for physicians, as knowing the survival period can inform treatment decisions and benefit patients in managing costs [112], [113], [114]. For most of the medical history, predictions relied primarily on the physician’s knowledge and experience based on prior patient histories and medical records. However, studies have indicated that physicians tend to execute poorly in predicting the prognosis and survival expectancy, often over-predicting survival time [115], [116], [117]. Statistical algorithms, such as the Cox proportional-hazards model [118], have been implemented to assist physicians’ prediction in many studies [119], [120], [121], [122], but they are not particularly accurate [12]. As a comparison, ML has shown its potential to predict a patient’s prognosis and survival in genomic, transcriptomic, proteomic, radiomic, and other datasets (Figure 4; Table 3). Chen et al. [123] used 3-year survival as a threshold to split the patients into high-risk (survival time < 36 months) and low-risk (survival time > 36 months) groups, and then constructed a NN model to binary predict the risk of a patient using his gene expression data and clinical variables. In their model, they tested four microarray gene expression datasets and achieved an overall accuracy of 83.0% with only five identified genes correlated with survival time. Liu et al. [124] also utilized gene expression data for a 3-year survival classification. Unlike Chen et al. [123], the authors integrated three types of sequencing data — RNA sequencing, DNA methylation, and DNA mutation — to select a total of 22 genes to improve their model’s stability. Meanwhile, LUADpp [125] and Cho et al. [126] used the somatic mutations as input features to model a 3-year survival risk classification. To select the genes associated with the highest significant mortality, Cho et al. [126] used chi-squared tests, and LUADpp [125] used a published genome-wide rate comparison test [127] that was able to balance statistical power and precision to compare gene mutation rates. Due to the complexity of survival prediction, multi-omics tumor data have been integrated for analysis in many studies. Compared with single-omics data, the multi-omics data are more challenging to accurately extract the most significant genes for prediction. To address the issue, several studies [128], [129], [130], [131] designed a similar workflow. They first constructed a matrix representing the similarity between patients based on their multi-omics data. Using the obtained matrix, they then employed an unsupervised clustering model (usually autoencoder with K-means clustering) to categorize the patients into two clusters. The two clusters were labeled “high-risk” and “low-risk” in terms of the different survival outcomes between the two clusters in the Kaplan–Meier analysis. Following the survival outcome differences, the genes associated with mortality were extracted using a statistical model [128], [129] or an ML model [130], [131] for downstream analyses.

## Apply ML to lung cancer immunotherapy

### Immunotherapy response prediction

Immunotherapy has become increasingly important in recent years. It enables a patient’s own immune system to fight cancer, in most cases, by stimulating T cells. Up to date, distinct novel immunotherapy treatments are being tested for lung cancer, and a variety of them have become standard parts of immunotherapy. Immune checkpoint inhibitors (ICIs), especially programmed cell death protein 1 (PD‐1)/programmed cell death protein ligand 1 (PD‐L1) blockade therapy [132], have been demonstrated to be valuable in the treatment of patients with non-small cell lung cancer (NSCLC) [133], [134]. However, immunotherapy is not yet as widely used as surgery, chemotherapy, or radiation therapies. One interpretation is that it does not work for all patients due to the uniqueness of a patient’s tumor immune microenvironment (TIME). Therefore, estimating whether a patient will respond to immunotherapy is important for cancer treatment. Recently, AI-based technologies have been developed to predict immunotherapy responses based on immune sequencing signatures and medical imaging signatures (Figure 4; Table 3) [135]. To predict the response to PD-1/PD-L1 blockade therapy, Wiesweg et al. [136] utilized gene expression profiles of 7 significant genes extracted from ML models plus 25 cell type-specific genes as input features to train an SVM classifier for RECIST classification. Aside from sequencing data, features from CT scans can also be used to assess the RECIST level of a patient. Two recent studies [137], [138] used radiomic biomarkers as well as other imaging features of tumor lesions from contrast-enhanced computed tomography (CE-CT) scans to train a classifier, including LR and RF, for RECIST classification.

### Tumor-infiltrating lymphocyte evaluation

The proportion of tumor-infiltrating lymphocytes (TILs) is another important metric for immunotherapy response evaluation. To this end, using transcriptomics data, DeepTIL [139] optimized the cell deconvolution model CIBERSORT [140] to automatically compute the abundance of the leucocyte subsets (B cells, CD4^+^ T cells, CD8^+^ T cells, γδ T cells, Mo-Ma-DC cells, and granulocytes) within a tumor sample. A different approach [141] utilized a total of 84 radiomic features from the CE-CT scans, along with RNA sequencing of 20,530 genes as biomarkers to train a linear elastic-net regression model to predict the abundance of CD8^+^ T cells. Another study [142] created a DL model to identify TILs in digitized H&E-stained images (Table 3). The methodology consisted of two unique CNN modules to evaluate TILs at different scales: a lymphocyte infiltration classification CNN (lymphocyte CNN) and a necrosis segmentation CNN (necrosis CNN). The “lymphocyte CNN” aimed to categorize the input image into with- and without-lymphocyte infiltration regions. It consists of two steps: a convolutional autoencoder (CAE) [143] for feature extraction, followed by a VGG 16-layer network [144] for TIL region classification. The “necrosis CNN” aimed to detect TILs within a necrosis region. They used the DeconvNet [145] model for TIL segmentation in “necrosis CNN” as the model has been shown to achieve high accuracy with several benchmark imaging datasets.

### Neoantigen prediction

In addition to immunotherapy response prediction, ML algorithms have shed light on neoantigen prediction for immunotherapy. Neoantigens are tumor-specific mutated peptides generated by somatic mutations in tumor cells, which can induce antitumor immune responses [146], [147], [148]. Recent work has demonstrated that immunogenic neoantigens are benefit to the development and optimization of neoantigen-targeted immune therapies [149], [150], [151], [152]. In accordance with neoantigen studies in clinical trials, state-of-the-art ML approaches have been implemented to identify neoantigens based on human leukocyte antigen (HLA) class I and II processing and presentation [153], [154], [155], [156], [157]. Using the identified somatic mutations, ML models can estimate the binding affinity of the encoded mutated peptides to the patient’s HLA alleles (peptide–HLA binding affinity). The neoantigens can be further predicted based on the estimated peptide–HLA binding affinity. NetMHC [158], [159] utilized a receptor–ligand dataset consisting of 528 peptide–HLA binding interactions measured by Buus et al. [160] to train a combination of several NNs for neo-peptide affinity prediction. To make the prediction more accurate, NetMHCpan [161], [162] used a larger dataset consisting of 37,384 unique peptide–HLA interactions covering 24 HLA-A alleles and 18 HLA-B alleles (26,503 and 10,881 for the A and B alleles, respectively) to train their NN model. Both tools have been implemented to study the neoantigen landscape in lung cancers [146], [163], [164], [165].

## Challenges and future perspectives

Despite the widespread use of ML studies in lung cancer clinical practice and research, there are still challenges to be addressed. Here, we post some examples of recent ML algorithms, especially the increasingly popular and important DL algorithms of the past decade, to enlighten them on lung cancer therapy analyses, as well as the challenges for future lung cancer studies.

### Imaging data analysis

Learning how to effectively extract nuance from imaging data is critical for clinical use. In the earlier ML-based CAD system, feature extractions were typically based on the image intensity, shape, and texture of a suspicious region along with other clinical variables [166]. However, these approaches are arbitrarily defined and may not retrieve the intrinsic features of a suspicious nodule. To this end, a DL-based CAD system was developed leveraging CNN models to extract features directly from raw imaging data with multilevel representations and hierarchical abstraction [167], [168], [169]. Contrary to previous methods, features from a CNN model are not designed by humans, and reflect the intrinsic features of the nodule in an objective and comprehensive manner. Recently, the Vision Transformer (ViT) has emerged as the current state-of-the-art in computer vision [170], [171]. In comparison to CNN, ViT outperformed almost 4× in terms of computational efficiency and accuracy, and was more robust when training on smaller datasets [172]. Although, to our knowledge, ViT models have not been implemented in any lung cancer imaging studies, they have shown their potential as a competitive alternative to CNN in imaging data analysis.

### Omics dataset analysis

DL is a subfield of ML, which uses programmable NNs to make accurate decisions. It particularly shines when it comes to complex problems such as image classification. In this study, we reviewed the utility of DL models in imaging datasets. Compared with imaging datasets, DL algorithms were less frequent in lung cancer clinical studies using omics data. However, DL models have been extensively applied in other fields of omics analysis. For example, the genomics data are continuous sequences, thus recurrent neural network (RNN) models [173] and CNN models [174] are good tools for the population genetics analysis. Moreover, considering the input dimension of the omics data is usually very high, to improve efficiency and reduce overfitting, many studies have used autoencoders or deep generative models for feature extraction and dimensionality reduction [175]. In the meantime, self-supervised representation learning models can overcome the curse of dimensionality and integrate multi-omics data to combine information about different aspects of the same tissue samples [176]. Accompanied by the development of single-cell-based [177] and spatial-based [178] technologies that have been applied in molecular studies, numerous DL models are becoming more popular for computationally intensive analysis. To deal with the complexity of large genomics data, unsupervised deep clustering tools have been built for population structure identification [179] or cell population subtype annotation [180], [181], [182], [183]. In addition, to process the complex structure of multi-omics data, graph neural network (GNN) models are increasingly popular in dataset integration [184], biomedical classification [185], prognosis prediction [186], and so on. Though these studies have not been directly applied to lung cancer clinical analysis, they are a good inspiration for using DL tools to address complex lung cancer omics datasets.

### Multi-view data and multi-database integration

It is common to access large amounts of imaging data, multi-omics data, and clinical records from a single patient nowadays. Integrating these data provides a comprehensive insight into the molecular functions of lung cancer studies. However, these data types are typically obtained from different platforms, so platform noise inevitably exists between these data types. For example, imaging data analysis, especially radiomics, usually comes with the challenges of complicated data normalization, data fusion, and data integration. To overcome this limitation, multimodality medical segmentation networks have been developed to jointly process multimodality medical images [187]. Similarly, for sequencing data types, batch noise also exists between different databases (*i.e.*, batch effect). Removing batch effects and integrating datasets from multiple platforms together in a framework that allows us to further analyze the mechanisms of cancer drug resistance and recurrence is important for cancer therapies. Though biomedical studies have experimented and/or benchmarked integrative tools [188], [189], [190], [191], they are not comprehensive and discriminating enough to address the choice of tools in the context of biological questions of interest.

### Model generalizability and robustness

In terms of this review, we find that the performance of an ML algorithm usually varies across different datasets. One interpretation might be the existence of a database batch effect that we discussed earlier. However, the absence of generalizability and robustness might be other factors that hurdle these ML models in clinical studies. In addition, to reduce overfitting, most studies used either statistical models or ML models to select marker genes before classification. However, these marker genes are usually quite different between studies, indicating that the identified marker genes lack generalizability and biological interpretability. To improve the generalizability and robustness of a model, it is important to develop a better understanding of robustness issues in different ML architectures and bridge the gap in robustness techniques among different domains. For example, recent studies have applied transfer learning to use a pre-trained model when training their own datasets in lung cancer imaging data analysis [38], [55], [192], and have improved the efficiency and robustness of their CNN-based models. For sequencing datasets, transfer learning has also been used in deep NNs to provide a generalizability approach [193], which could be a good example of building a general and robust model for lung cancer sequencing data analysis. In addition, DL is a complex black-box model. Understanding the mechanisms of a DL system in clinical studies could help to build a standardized and unified DL framework to improve its performance and robustness. The explainable AI (XAI) models have provided a tool for model-specific and model-agnostic analysis [194], [195]. These methods can provide the explanations of a model at local and global levels, which further helps the researchers to fine-tune hyper-parameters from different models with high efficacy [196], [197].

### Metrics for performance evaluation

Studies usually focus on the development of algorithms for clinical studies. However, metrics selection for performance assessment of these algorithms is usually neglected, though it usually plays an important component in ML systems [198]. Based on this review (Table 1, Table 2, Table 3), accuracy and under the curve (AUC) are the two most conventional metrics, whereas these metrics do not always reflect the clinical needs and should be translated into clinically explainable metrics. Compared with accuracy, sensitivity or specificity might be more associated with clinical needs under certain circumstances, for example, patients at high risk of emergency department visits [199].

### Clinical decision-making

A recent study estimated that the overall costs for lung cancer therapy would exceed $50,000 [200] for most patients, and that the cost would be high for most families. Thus, accurate prognosis prediction and decision-making will pave the way for personalized treatment. Recent DL models have been used to predict the effectiveness of a therapy/drug and optimize the combination of different therapies/drugs [201], [202]. However, most existing DL models for clinical decision-making have difficulty in keeping up with knowledge evolution and/or dynamic health care data change [203]. Currently, clinical decision support systems, including IBM Watson Health and Google DeepMind Health, have been implemented in lung cancer treatments in recent years [204], [205]. Although the efficiency of clinical work has improved with the help of these systems, they are still far from perfect in terms of clinical trials, and currently cannot replace physicians at this stage [205].

## Conclusion

AI grants us a different perspective on lung cancer research and allows for exploring the implementation of decision support tools to facilitate precision oncology. In this review, we surveyed the current advances of ML algorithms in various areas of lung cancer therapy, including early detection, diagnosis decision, prognosis prediction, drug response evaluation, and immunotherapy practice. To aid future ML development in lung cancer therapies, we thoroughly summarized the datasets (Table S1), baseline methods (Table S2), and characteristics of the methods (Table 1, Table 2, Table 3). At last, we highlighted the current challenges that need to be addressed, such as the current lack of quantity and quality of medical data labels for training, the importance of model robustness and biomedical explanations for clinical use, the concern of the metrics used for performance evaluation, and the need for data integration and batch removal. As this review indicates, future lung cancer therapies will include both imaging data and omics data, so an ML clinical decision-making tool should be a multi-modal system that considers both imaging data and omics data treatment, and the integration of multiple data types. Finally, we expect that these challenges could motivate further studies to focus on lung cancer therapies.

## CRediT author statement

**Yawei Li:** Conceptualization, Data curation, Writing - original draft, Visualization. **Xin Wu:** Data curation, Writing - original draft, Visualization. **Ping Yang:** Writing - review & editing. **Guoqian Jiang:** Writing - review & editing. **Yuan Luo:** Conceptualization, Funding acquisition, Writing - review & editing. All authors have read and approved the final manuscript.

## Competing interests

The authors have declared no competing interests.

## Supplementary material

Supplementary Table S1 **Lung cancer benchmark datasets used by the machine learning methods reviewed in this paper**

Supplementary Table S2 **Machine learning methods used for benchmark studies in lung cancer therapy**
