# Supplementary material for: Machine Learning for Lung Cancer Diagnosis, Treatment, and Prognosis
Source: Genomics Proteomics Bioinformatics. 2022 Dec 1;20(5):850–66. doi: 10.1016/j.gpb.2022.11.003 (PMC10025752; doi:10.1016/j.gpb.2022.11.003)
Supplement: Supplementary Table S1 — Lung cancer benchmark datasets used by the machine learning methods reviewed in this paper [file mmc1.docx]

**Table S1 Lung cancer benchmark datasets used by the machine learning methods reviewed in this paper**

| **No.** | **Database** | **Data type** | **Website** | **Ref.** |
| --- | --- | --- | --- | --- |
| 1 | TCGA | Genomics data | https://www.cancer.gov/about-nci/organization/ccg/research/structural-genomics/tcga | [1] |
| 2 | TCIA | Image (CT, MRI, PET, *etc.*) | https://www.cancerimagingarchive.net/ | [2] |
| 3 | PanCan | CT | https://www.thelancet.com/journals/lanonc/article/PIIS1470-2045(17)30597-1/fulltext | [3] |
| 4 | BCCA | CT | https://www.atsjournals.org/doi/full/10.1164/rccm.200301-144OC | [4] |
| 5 | DLCST | CT | https://www.sciencedirect.com/science/article/pii/S1556086415316786 | [5] |
| 6 | Kriegsmann et al. | MALDI | https://www.ncbi.nlm.nih.gov/pmc/articles/PMC5054336/ | [6] |
| 7 | LIDC-IDRI | CT | https://wiki.cancerimagingarchive.net/display/Public/LIDC-IDRI | [7] |
| 8 | LTRC | CT | https://ltrcpublic.com/ |  |
| 9 | NLST | CT | https://www.cancer.gov/types/lung/research/nlst |  |
| 10 | NELSON | CT | https://acsjournals.onlinelibrary.wiley.com/doi/10.1002/cncr.23590 | [8] |
| 11 | Venkadesh et al. | CT | https://pubs.rsna.org/doi/full/10.1148/radiol.2021204433 | [9] |
| 12 | Jiang et al. | CT | https://ieeexplore.ieee.org/document/8417454 | [10] |
| 13 | TRACERx | Histological image | https://www.nejm.org/doi/10.1056/NEJMoa1616288?url_ver=Z39.88-2003&rfr_id=ori:rid:crossref.org&rfr_dat=cr_pub%20%200www.ncbi.nlm.nih.gov | [11] |
| 14 | TCGA–LUSC | Histological image;  Genomics | https://www.nature.com/articles/nature11404 | [12] |
| 15 | TCGA–LUAD | Histological image;  Genomics | https://www.nature.com/articles/nature13385 | [13] |
| 16 | Pan-Lung Cancer dataset | Exome sequences and copy number profiles | https://www.nature.com/articles/ng.3564#Sec20 | [14] |
| 17 | AEGIS | RNA | https://www.ncbi.nlm.nih.gov/geo/query/acc.cgi?acc=GSE4115 | [15] |
| 18 | Bhattacharjee et al. | RNA | https://www.pnas.org/content/98/24/13790.long | [16] |
| 19 | Beer et al. | RNA | https://www.nature.com/articles/nm733#Sec3 | [17] |
| 20 | Wigle et al. | RNA | https://cancerres.aacrjournals.org/content/62/11/3005.long | [18] |
| 21 | Gordon et al. | RNA | https://cancerres.aacrjournals.org/content/62/17/4963.long | [19] |
| 22 | Silvestri et al. | RNA | https://www.nejm.org/doi/full/10.1056/NEJMoa1504601 | [20] |
| 23 | Aliferis et al. | Array CGH | https://www.ncbi.nlm.nih.gov/pmc/articles/PMC2244172/pdf/procamiasymp00001-0048.pdf | [21] |
| 24 | Raman et al. | cfDNA | https://genomemedicine.biomedcentral.com/articles/10.1186/s13073-020-00735-4#Sec2 | [22] |
| 25 | CLCGP project | Genomics data | https://www.science.org/doi/10.1126/scitranslmed.3006802 | [23] |
| 26 | Daemen et al. | Array CGH | http://psb.stanford.edu/psb-online/proceedings/psb09/daemen.pdf | [24] |
| 27 | NCI caArray database | RNA | https://wiki.nci.nih.gov/display/caArray2/caArray+Retirement+Announcement | [25] |
| 28 | Wang et al. | Somatic mutation in EGFR | https://www.nature.com/articles/srep02855#Sec9 | [26] |
| 29 | Lee et al. | Genomic DNA in EGFR | https://www.sciencedirect.com/science/article/pii/S1556086415334705?via%3Dihub#bib18 | [27] |
| 30 | EGFR Mutation Database | Somatic mutation in EGFR | The data are available on request to the corresponding author. | [28] |
| 31 | Zou et al. | Somatic mutation in EGFR | https://www.nature.com/articles/s41598-017-06632-y#Sec11 | [29] |
| 32 | Garnett et al. | RNA | https://www.nature.com/articles/nature11005#ethics | [30] |
| 33 | Wiesweg et al. | RNA | https://www.sciencedirect.com/science/article/pii/S0959804920305219?via%3Dihub | [31] |
| 34 | Trebeschi et al. | CT | https://www.sciencedirect.com/science/article/pii/S0923753419312025?via%3Dihub | [32] |
| 35 | Coroller et al. | CT | https://www.sciencedirect.com/science/article/pii/S0167814016310386?via%3Dihub | [33] |
| 36 | GEO | RNA | https://www.ncbi.nlm.nih.gov/gds |  |
| 37 | MOSCATO database | CT, RNA | https://cancerdiscovery.aacrjournals.org/content/7/6/586.long#sec-8 | [34] |
| 38 | Champiat et al. | CT | https://clincancerres.aacrjournals.org/content/23/8/1920.long#sec-6 | [35] |
| 39 | Sun et al. | CT | https://www.sciencedirect.com/science/article/pii/S095980491731153X | [36] |
| 40 | Buus et al. | Tumor peptidomics dataset | https://www.sciencedirect.com/science/article/pii/030441659400172T?via%3Dihub | [37] |
| 41 | Peters et al. | Tumor peptidomics dataset | https://journals.plos.org/ploscompbiol/article?id=10.1371/journal.pcbi.0020065 | [38] |
| 42 | Bulik-Sullivan et al. | Tumor peptidomics dataset | https://massive.ucsd.edu/ProteoSAFe/dataset.jsp?task=ad676ada8227478e92996c2ef849ea31 | [39] |
| 43 | Mathios et al. | cfDNA | https://ega-archive.org/studies/EGAS00001005340 | [40] |
| 44 | Chabon et al. | cfDNA | https://clip.stanford.edu/ | [41] |
| 45 | Liang et al. | DNA methylation | https://www.thno.org/v09p2056.htm | [42] |
| 46 | Jurmeister et al. | DNA methylation | https://www.ncbi.nlm.nih.gov/geo/query/acc.cgi?acc=GSE124052 | [43] |
| 47 | SPIE-AAPM | CT | https://wiki.cancerimagingarchive.net/display/Public/SPIE-AAPM+Lung+CT+Challenge | [44] |
| 48 | Borkowski et al. | Histological image | https://www.kaggle.com/datasets/andrewmvd/lung-and-colon-cancer-histopathological-images | [45] |

*Note*: TCGA, The Cancer Genome Atlas; TCIA, The Cancer Imaging Archive; MRI, magnetic resonance imaging; PET, positron emission tomography; PanCan, Pan-Canadian Early Detection of Lung Cancer Study; BCCA, British Columbia Cancer Agency study; DLCST, the Danish Lung Cancer Screening Trial; LIDC-IDRI, Lung Image Database Consortium image collection; LTRC, Lung Tissue Research Consortium; NLST, National Lung Screening Trial; NELSON, Dutch-Belgian randomized lung cancer screening trial; MOSCATO, the Molecular Screening for Cancer Treatment Optimization; LUAD, lung adenocarcinoma; LUSC, lung squamous cell carcinoma; CLCGP, the Clinical Lung Cancer Genome Project; NCI, National Cancer Institute; GEO, Gene Expression Omnibus.

**References**

[1] Cancer Genome Atlas Research Network, Weinstein JN, Collisson EA, Mills GB, Shaw KR, Ozenberger BA, et al. The Cancer Genome Atlas Pan-Cancer analysis project. Nat Genet 2013;45:1113–20.

[2] Clark K, Vendt B, Smith K, Freymann J, Kirby J, Koppel P, et al. The Cancer Imaging Archive (TCIA): maintaining and operating a public information repository. J Digit Imaging 2013;26:1045–57.

[3] Tammemagi MC, Schmidt H, Martel S, McWilliams A, Goffin JR, Johnston MR, et al. Participant selection for lung cancer screening by risk modelling (the Pan-Canadian Early Detection of Lung Cancer [PanCan] study): a single-arm, prospective study. Lancet Oncol 2017;18:1523–31.

[4] McWilliams A, Mayo J, MacDonald S, leRiche JC, Palcic B, Szabo E, et al. Lung cancer screening: a different paradigm. Am J Respir Crit Care Med 2003;168:1167–73.

[5] Pedersen JH, Ashraf H, Dirksen A, Bach K, Hansen H, Toennesen P, et al. The Danish randomized lung cancer CT screening trial — overall design and results of the prevalence round. J Thorac Oncol 2009;4:608–14.

[6] Kriegsmann M, Casadonte R, Kriegsmann J, Dienemann H, Schirmacher P, Kobarg JH, et al. Reliable entity subtyping in non-small cell lung cancer by matrix-assisted laser desorption/ionization imaging mass spectrometry on formalin-fixed paraffin-embedded tissue specimens. Mol Cell Proteomics 2016;15:3081–9.

[7] Armato SG 3rd, McLennan G, Bidaut L, McNitt-Gray MF, Meyer CR, Reeves AP, et al. The Lung Image Database Consortium (LIDC) and Image Database Resource Initiative (IDRI): a completed reference database of lung nodules on ct scans. Med Phys 2011;38:915–31.

[8] van den Bergh KAM, Essink-Bot M-L, Bunge EM, Scholten ET, Prokop M, van Iersel CA, et al. Impact of computed tomography screening for lung cancer on participants in a randomized controlled trial (NELSON trial). Cancer 2008;113:396–404.

[9] Venkadesh KV, Setio AAA, Schreuder A, Scholten ET, Chung KM, Wille MMW, et al. Deep learning for malignancy risk estimation of pulmonary nodules detected at low-dose screening CT. Radiology 2021;300:438–47.

[10] Jiang J, Hu YC, Liu CJ, Halpenny D, Hellmann MD, Deasy JO, et al. Multiple resolution residually connected feature streams for automatic lung tumor segmentation from CT images. IEEE Trans Med Imaging 2019;38:134–44.

[11] Jamal-Hanjani M, Wilson GA, McGranahan N, Birkbak NJ, Watkins TBK, Veeriah S, et al. Tracking the evolution of non-small-cell lung cancer. N Engl J Med 2017;376:2109–21.

[12] Cancer Genome Atlas Research Network. Comprehensive genomic characterization of squamous cell lung cancers. Nature 2012;489:519–25.

[13] Cancer Genome Atlas Research Network. Comprehensive molecular profiling of lung adenocarcinoma. Nature 2014;511:543–50.

[14] Campbell JD, Alexandrov A, Kim J, Wala J, Berger AH, Pedamallu CS, et al. Distinct patterns of somatic genome alterations in lung adenocarcinomas and squamous cell carcinomas. Nat Genet 2016;48:607–16.

[15] Spira A, Beane JE, Shah V, Steiling K, Liu G, Schembri F, et al. Airway epithelial gene expression in the diagnostic evaluation of smokers with suspect lung cancer. Nat Med 2007;13:361–6.

[16] Bhattacharjee A, Richards WG, Staunton J, Li C, Monti S, Vasa P, et al. Classification of human lung carcinomas by mRNA expression profiling reveals distinct adenocarcinoma subclasses. Proc Natl Acad Sci U S A 2001;98:13790–5.

[17] Beer DG, Kardia SL, Huang CC, Giordano TJ, Levin AM, Misek DE, et al. Gene-expression profiles predict survival of patients with lung adenocarcinoma. Nat Med 2002;8:816–24.

[18] Wigle DA, Jurisica I, Radulovich N, Pintilie M, Rossant J, Liu N, et al. Molecular profiling of non-small cell lung cancer and correlation with disease-free survival. Cancer Res 2002;62:3005–8.

[19] Gordon GJ, Jensen RV, Hsiao LL, Gullans SR, Blumenstock JE, Ramaswamy S, et al. Translation of microarray data into clinically relevant cancer diagnostic tests using gene expression ratios in lung cancer and mesothelioma. Cancer Res 2002;62:4963–7.

[20] Silvestri GA, Vachani A, Whitney D, Elashoff M, Porta Smith K, Ferguson JS, et al. A bronchial genomic classifier for the diagnostic evaluation of lung cancer. N Engl J Med 2015;373:243–51.

[21] Aliferis CF, Hardin D, Massion PP. Machine learning models for lung cancer classification using array comparative genomic hybridization. Proc AMIA Symp 2002:7–11.

[22] Raman L, van der Linden M, van der Eecken K, Vermaelen K, Demedts I, Surmont V, et al. Shallow whole-genome sequencing of plasma cell-free DNA accurately differentiates small from non-small cell lung carcinoma. Genome Med 2020;12:35.

[23] Clinical Lung Cancer Genome Project, Network Genomic Medicine. A genomics-based classification of human lung tumors. Sci Transl Med 2013;5:209ra153.

[24] Daemen A, Gevaert O, Leunen K, Legius E, Vergote I, De Moor B. Supervised classification of array CGH data with HMM-based feature selection. Pac Symp Biocomput 2009:468–79.

[25] Director’s Challenge Consortium for the Molecular Classification of Lung Adenocarcinoma, Shedden K, Taylor JMG, Enkemann SA, Tsao MS, Yeatman TJ, et al. Gene expression-based survival prediction in lung adenocarcinoma: a multi-site, blinded validation study. Nat Med 2008;14:822–7.

[26] Wang DD, Zhou W, Yan H, Wong M, Lee V. Personalized prediction of EGFR mutation-induced drug resistance in lung cancer. Sci Rep 2013;3:2855.

[27] Lee VHF, Tin VP, Choy TS, Lam KO, Choi CW, Chung LP, et al. Association of exon 19 and 21 EGFR mutation patterns with treatment outcome after first-line tyrosine kinase inhibitor in metastatic non-small-cell lung cancer. J Thorac Oncol 2013;8:1148–55.

[28] Gu D, Scaringe WA, Li K, Saldivar JS, Hill KA, Chen Z, et al. Database of somatic mutations in EGFR with analyses revealing indel hotspots but no smoking-associated signature. Hum Mutat 2007;28:760–70.

[29] Zou B, Lee VHF, Chen L, Ma L, Wang DD, Yan H. Deciphering mechanisms of acquired T790M mutation after EGFR inhibitors for NSCLC by computational simulations. Sci Rep 2017;7:6595.

[30] Garnett MJ, Edelman EJ, Heidorn SJ, Greenman CD, Dastur A, Lau KW, et al. Systematic identification of genomic markers of drug sensitivity in cancer cells. Nature 2012;483:570–5.

[31] Wiesweg M, Mairinger F, Reis H, Goetz M, Kollmeier J, Misch D, et al. Machine learning reveals a PD-L1-independent prediction of response to immunotherapy of non-small cell lung cancer by gene expression context. Eur J Cancer 2020;140:76–85.

[32] Trebeschi S, Drago SG, Birkbak NJ, Kurilova I, Calin AM, Pizzi AD, et al. Predicting response to cancer immunotherapy using noninvasive radiomic biomarkers. Ann Oncol 2019;30:998–1004.

[33] Coroller TP, Agrawal V, Narayan V, Hou Y, Grossmann P, Lee SW, et al. Radiomic phenotype features predict pathological response in non-small cell lung cancer. Radiother Oncol 2016;119:480–6.

[34] Massard C, Michiels S, Ferte C, Le Deley MC, Lacroix L, Hollebecque A, et al. High-throughput genomics and clinical outcome in hard-to-treat advanced cancers: results of the MOSCATO 01 trial. Cancer Discov 2017;7:586–95.

[35] Champiat S, Dercle L, Ammari S, Massard C, Hollebecque A, Postel-Vinay S, et al. Hyperprogressive disease is a new pattern of progression in cancer patients treated by anti-PD-1/PD-L1. Clin Cancer Res 2017;23:1920–8.

[36] Sun R, Champiat S, Dercle L, Aspeslagh S, Castanon E, Limkin EJ, et al. Baseline lymphopenia should not be used as exclusion criteria in early clinical trials investigating immune checkpoint blockers (PD-1/PD-L1 inhibitors). Eur J Cancer 2017;84:202–11.

[37] Buus S, Stryhn A, Winther K, Kirkby N, Pedersen LO. Receptor-ligand interactions measured by an improved spun column chromatography technique. a high efficiency and high throughput size separation method. Biochim Biophys Acta 1995;1243:453–60.

[38] Peters B, Bui H-H, Frankild S, Nielson M, Lundegaard C, Kostem E, et al. A community resource benchmarking predictions of peptide binding to MHC-I molecules. PLoS Comput Biol 2006;2:e65.

[39] Bulik-Sullivan B, Busby J, Palmer CD, Davis MJ, Murphy T, Clark A, et al. Deep learning using tumor HLA peptide mass spectrometry datasets improves neoantigen identification. Nat Biotechnol 2019;37:55–63.

[40] Mathios D, Johansen JS, Cristiano S, Medina JE, Phallen J, Larsen KR, et al. Detection and characterization of lung cancer using cell-free DNA fragmentomes. Nat Commun 2021;12:5060.

[41] Chabon JJ, Hamilton EG, Kurtz DM, Esfahani MS, Moding EJ, Stehr H, et al. Integrating genomic features for non-invasive early lung cancer detection. Nature 2020;580:245–51.

[42] Liang W, Zhao Y, Huang W, Gao Y, Xu W, Tao J, et al. Non-invasive diagnosis of early-stage lung cancer using high-throughput targeted DNA methylation sequencing of circulating tumor DNA (ctDNA). Theranostics 2019;9:2056–70.

[43] Jurmeister P, Bockmayr M, Seegerer P, Bockmayr T, Treue D, Montavon G, et al. Machine learning analysis of DNA methylation profiles distinguishes primary lung squamous cell carcinomas from head and neck metastases. Sci Transl Med 2019;11:eaaw8513.

[44] Armato III SG, Hadjiiski LM, Tourassi GD, Drukker K, Giger ML, Li F, et al. SPIE-AAPM-NCI lung nodule classification challenge dataset. [Internet]. https://wiki.cancerimagingarchive.net/display/Public/SPIE-AAPM+Lung+CT+Challenge.

[45] Borkowski AA, Bui MM, Thomas LB, Wilson CP, DeLand LA, Mastorides SM. Lung and colon cancer histopathological image dataset (LC25000). arXiv 2019; https://doi.org/10.48550/arXiv.1912.12142.
