# Supplementary material for: Machine Learning for Lung Cancer Diagnosis, Treatment, and Prognosis
Source: Genomics Proteomics Bioinformatics. 2022 Dec 1;20(5):850–66. doi: 10.1016/j.gpb.2022.11.003 (PMC10025752; doi:10.1016/j.gpb.2022.11.003)
Supplement: Supplementary Table S2 — Machine learning methods used for benchmark studies in lung cancer therapy [file mmc2.docx]

**Table S2 Machine learning methods used for benchmark studies in lung cancer therapy**

| **Name** | **Application scenarios** | **Datasets in Table S1** | **Benchmarks** | **Ref.** |
| --- | --- | --- | --- | --- |
| McWilliams et al. | ML on early detection and diagnosis using medical imaging datasets | 3, 4 | No | [1] |
| Riel et al. | ML on early detection and diagnosis using medical imaging datasets | 5 | Radiologists | [2] |
| Wille et al. | ML on early detection and diagnosis using medical imaging datasets | 3, 4, 5 | No | [3] |
| Kriegsmann et al. | ML on early detection and diagnosis using medical imaging datasets | 6 | No | [4] |
| Buty et al. | ML on early detection and diagnosis using medical imaging datasets | 7 | No | [5] |
| Hussein et al. | ML on early detection and diagnosis using medical imaging datasets | 7 | GIST features [6] with LASSO; 3D CNN-based (Karpathy et al. [7]) multi-task learning with trace norm | [8] |
| Khosravan et al. | ML on early detection and diagnosis using medical imaging datasets | 8 | Khosravan et al. [9]; Dou et al. [10]; Radiologists | [11] |
| Ciompi et al. | ML on early detection and diagnosis using medical imaging datasets | 10 | RF; SVM; Radiologists | [12] |
| Venkadesh et al. | ML on early detection and diagnosis using medical imaging datasets | 11 | Radiologists; PanCan model [13] | [14] |
| Ardila et al. | ML on early detection and diagnosis using medical imaging datasets | 7, 9 | No | [15] |
| AbdulJabbar et al. | ML on early detection and diagnosis using medical imaging datasets | 13 | No | [16] |
| Coudray et al. | ML on early detection and diagnosis using medical imaging datasets | 14, 15 | No | [17] |
| Lin et al. | ML on early detection and diagnosis using medical imaging datasets | 47 | No | [18] |
| Ren et al. | ML on early detection and diagnosis using medical imaging datasets | 48 | ResNet50, DenseNet121, and EfficientNetB4 | [19] |
| Mathios et al. | ML on early detection and diagnosis using -omics sequencing datasets | 1, 43 | No | [20] |
| Lung-CLiP | ML on early detection and diagnosis using -omics sequencing datasets | 44 | 5-nearest neighbor; 3-nearest neighbor; NB; LR; DT | [21] |
| Liang et al. | ML on early detection and diagnosis using -omics sequencing datasets | 45 | No | [22] |
| Raman et al. | ML on early detection and diagnosis using -omics sequencing datasets | 24, 25 | RF; SVM; LR with ridge; Elastic Net [23]; LASSO regularization [24] | [25] |
| Kobayashi et al. | ML on early detection and diagnosis using -omics sequencing datasets | 16 | MLP; Diet Networks [26] | [27] |
| Whitney et al. | ML on early detection and diagnosis using -omics sequencing datasets | 17 | No | [28] |
| Podolsky et al. | ML on early detection and diagnosis using -omics sequencing datasets | 18, 19, 20,21 | KNN; NB; SVM; DT | [29] |
| Choi et al. | ML on early detection and diagnosis using -omics sequencing datasets | 22 | RF; SVM; LDA; GB; penalized LR | [30] |
| Aliferis et al. | ML on early detection and diagnosis using -omics sequencing datasets | 18 | linear SVM; polynomial-kernel SVM; KNN; NN | [31] |
| Aliferis et al. | ML on early detection and diagnosis using -omics sequencing datasets | 23 | DT; KNN; linear SVM; polynomial-kernel SVM; RBF-kernel SVM; NN | [32] |
| Daemen et al. | ML on early detection and diagnosis using -omics sequencing datasets | 26 | No | [33] |
| Jurmeister et al. | ML on early detection and diagnosis using -omics sequencing datasets | 46 | NN; SVM; RF | [34] |
| Jiang et al. | Prognosis and therapy response prediction | 2, 7, 12 | FRRN [35]; Unet [36]; SegNet [37]; RF+fCRF [38] | [39] |
| Qureshi | Prognosis and therapy response prediction | 28, 29, 30, 31 | Wang et al. [40]; Ma et al. [41]; Duan et al. [42]; Zou et al. [43]; Kureshi et al. [44] | [45] |
| Kapil et al. | Prognosis and therapy response prediction | Not publicly available | Shallow VGG [46], inception network v2 [47] | [48] |
| Geeleher et al. | Prognosis and therapy response prediction | 32 | RF; PAM [49]; Principal component regression [50]; Lasso regression [24]; Elastic Net regression [23] | [51] |
| Chen et al. | Survival prediction | 27 | SVM; Bayesian; KNN | [52] |
| Liu et al. | Survival prediction | 1, 36 | SVM; RF; LR: NB; linear regression; SVR (kernel Poly); SVR (kernel Linear); ridge regression | [53] |
| LUADpp | Survival prediction | 1 | No | [54] |
| Cho et al. | Survival prediction | 1 | NB; KNN; SVM; DT | [55] |
| Yu et al. | Survival prediction | 1 | No | [56] |
| CIMLR | Survival prediction | 1 | iCluster+ [57]; Bayesian consensus clustering [58]; PINS [59]; SNF [60] | [61] |
| Asada et al. | Survival prediction | 1 | SVM; KNN; RF; LR | [62] |
| Takahashi et al. | Survival prediction | 1 | No | [63] |
| Wiesweg et al. | Immunotherapy response prediction | 33 | RF, LR, XGBoost | [64] |
| Trebeschi et al. | Immunotherapy response prediction | 34 | No | [65] |
| Coroller et al. | Immunotherapy response prediction | 35 | No | [66] |
| DeepTIL | Tumor-infiltrating lymphocytes (TILs) evaluation | 36 | CIBERSORT [67] | [68] |
| Sun et al. | Tumor-infiltrating lymphocytes (TILs) evaluation | 1, 2, 37, 38, 39 | No | [69] |
| Saltz et al. | Tumor-infiltrating lymphocytes (TILs) evaluation | 1 | Zhao et al. [70] | [71] |
| Bulik-Sullivan et al. | Neoantigen prediction | 42 | NetMHC [72]; MHCflurry [73]; NetMHCpan [74] | [75] |
| NetMHC | Neoantigen prediction | 40 | No | [72] |
| NetMHC-pan | Neoantigen prediction | 41 | NetMHC [72] | [74] |

*Note*: GIST, gastrointestinal stromal tumors; MLP, multilayer perceptron; GB, gradient boosting; RF+fCRF, random forest with fully connected conditional random field; SVR, support vector regression.

**References**

[1] McWilliams A, Tammemagi MC, Mayo JR, Roberts H, Liu G, Soghrati K, et al. Probability of cancer in pulmonary nodules detected on first screening CT. N Engl J Med 2013;369:910–9.

[2] van Riel SJ, Ciompi F, Winkler Wille MM, Dirksen A, Lam S, Scholten ET, et al. Malignancy risk estimation of pulmonary nodules in screening CTs: comparison between a computer model and human observers. PLoS One 2017;12:e0185032.

[3] Wille MMW, van Riel SJ, Saghir Z, Dirksen A, Pedersen JH, Jacobs C, et al. Predictive accuracy of the pancan lung cancer risk prediction model -external validation based on CT from the danish lung cancer screening trial. Eur Radiol 2015;25:3093–9.

[4] Kriegsmann M, Casadonte R, Kriegsmann J, Dienemann H, Schirmacher P, Kobarg JH, et al. Reliable entity subtyping in non-small cell lung cancer by matrix-assisted laser desorption/ionization imaging mass spectrometry on formalin-fixed paraffin-embedded tissue specimens. Mol Cell Proteomics 2016;15:3081–9.

[5] Buty M, Xu Z, Gao M, Bagci U, Wu A, Mollura D. Characterization of lung nodule malignancy using hybrid shape and appearance features. In: Ourselin S, Joskowicz L, Sabuncu M, Unal G, Wells W, editors. Medical image computing and computer-assisted intervention. Cham: Springer; 2016, p.662–70.

[6] Oliva A, Torralba A. Modeling the shape of the scene: a holistic representation of the spatial envelope. Int J Comput Vis 2001;42:145–75.

[7] Karpathy A, Toderici G, Shetty S, Leung T, Sukthankar R, Li FF. Large-scale video classification with convolutional neural networks. IEEE Conf Comput Vis Pattern Recognit 2014:1725–32.

[8] Hussein S, Cao K, Song Q, Bagci U. Risk stratification of lung nodules using 3D CNN-based multi-task learning. In: Niethammer M, Styner M, Aylward S, Zhu H, Oguz I, Yap P-T, et al, editors. Information processing in medical imaging. Cham: Springer; 2017, p.249–60.

[9] Khosravan N, Celik H, Turkbey B, Cheng R, McCreedy E, McAuliffe M, et al. Gaze2Segment: a pilot study for integrating eye-tracking technology into medical image segmentation. In: Müller H, Kelm BM, Arbel T, Cai W, Cardoso MJ, Langs G, et al, editors. Medical computer vision and Bayesian and graphical models for biomedical imaging. Cham: Springer; 2017, p.94–104.

[10] Dou Q, Chen H, Yu L, Qin J, Heng PA. Multilevel contextual 3-D CNNs for false positive reduction in pulmonary nodule detection. IEEE Trans Biomed Eng 2017;64:1558–67.

[11] Khosravan N, Celik H, Turkbey B, Jones EC, Wood B, Bagci U. A collaborative computer aided diagnosis (C-CAD) system with eye-tracking, sparse attentional model, and deep learning. Med Image Anal 2019;51:101–15.

[12] Ciompi F, de Hoop B, van Riel SJ, Chung K, Scholten ET, Oudkerk M, et al. Automatic classification of pulmonary peri-fissural nodules in computed tomography using an ensemble of 2D views and a convolutional neural network out-of-the-box. Med Image Anal 2015;26:195–202.

[13] Tammemagi MC, Schmidt H, Martel S, McWilliams A, Goffin JR, Johnston MR, et al. Participant selection for lung cancer screening by risk modelling (the Pan-Canadian Early Detection of Lung Cancer [PanCan] study): a single-arm, prospective study. Lancet Oncol 2017;18:1523–31.

[14] Venkadesh KV, Setio AAA, Schreuder A, Scholten ET, Chung KM, Wille MMW, et al. Deep learning for malignancy risk estimation of pulmonary nodules detected at low-dose screening CT. Radiology 2021;300:438–47.

[15] Ardila D, Kiraly AP, Bharadwaj S, Choi B, Reicher JJ, Peng L, et al. End-to-end lung cancer screening with three-dimensional deep learning on low-dose chest computed tomography. Nat Med 2019;25:954–61.

[16] AbdulJabbar K, Raza SEA, Rosenthal R, Jamal-Hanjani M, Veeriah S, Akarca A, et al. Geospatial immune variability illuminates differential evolution of lung adenocarcinoma. Nat Med 2020;26:1054–62.

[17] Ocampo P, Moreira A, Coudray N, Sakellaropoulos T, Narula N, Snuderl M, et al. Classification and mutation prediction from non-small cell lung cancer histopathology images using deep learning. J Thorac Oncol 2018;13:S562.

[18] Lin CH, Lin CJ, Li YC, Wang SH. Using generative adversarial networks and parameter optimization of convolutional neural networks for lung tumor classification. Appl Sci 2021;11:480.

[19] Ren Z, Zhang Y, Wang S. A hybrid framework for lung cancer classification. Electronics 2022;11:1614.

[20] Mathios D, Johansen JS, Cristiano S, Medina JE, Phallen J, Larsen KR, et al. Detection and characterization of lung cancer using cell-free DNA fragmentomes. Nat Commun 2021;12:5060.

[21] Chabon JJ, Hamilton EG, Kurtz DM, Esfahani MS, Moding EJ, Stehr H, et al. Integrating genomic features for non-invasive early lung cancer detection. Nature 2020;580:245–51.

[22] Liang W, Zhao Y, Huang W, Gao Y, Xu W, Tao J, et al. Non-invasive diagnosis of early-stage lung cancer using high-throughput targeted DNA methylation sequencing of circulating tumor DNA (ctDNA). Theranostics 2019;9:2056–70.

[23] Zou H, Hastie T. Regularization and variable selection via the elastic net. Journal of the Royal Statistical Society. Series B (Statistical Methodology) 2005;67:301–20.

[24] Tibshirani R. Regression shrinkage and selection via the lasso. J R Stat Soc B 1996;58:267–88.

[25] Raman L, van der Linden M, van der Eecken K, Vermaelen K, Demedts I, Surmont V, et al. Shallow whole-genome sequencing of plasma cell-free DNA accurately differentiates small from non-small cell lung carcinoma. Genome Med 2020;12:35.

[26] Romero A, Carrier PL, Erraqabi A, Sylvain T, Auvolat A, Dejoie E, et al. Diet networks: Thin parameters for fat genomic. arXiv 2016; https://doi.org/10.48550/arXiv.1611.09340.

[27] Kobayashi K, Bolatkan A, Shiina S, Hamamoto R. Fully-connected neural networks with reduced parameterization for predicting histological types of lung cancer from somatic mutations. Biomolecules 2020;10:1249.

[28] Whitney DH, Elashoff MR, Porta-Smith K, Gower AC, Vachani A, Ferguson JS, et al. Derivation of a bronchial genomic classifier for lung cancer in a prospective study of patients undergoing diagnostic bronchoscopy. BMC Med Genomics 2015;8:18.

[29] Podolsky MD, Barchuk AA, Kuznetcov VI, Gusarova NF, Gaidukov VS, Tarakanov SA. Evaluation of machine learning algorithm utilization for lung cancer classification based on gene expression levels. Asian Pac J Cancer Prev 2016;17:835–8.

[30] Choi Y, Qu J, Wu S, Hao Y, Zhang J, Ning J, et al. Improving lung cancer risk stratification leveraging whole transcriptome RNA sequencing and machine learning across multiple cohorts. BMC Med Genomics 2020;13:151.

[31] Aliferis CF, Tsamardinos I, Massion PP, Statnikov A, Fananapazir N, Hardin D. Machine learning models for classification of lung cancer and selection of genomic markers using array gene expression data. Proc 16th Int Florida Artif Intell Res Soc Conf 2003:67–71.

[32] Aliferis CF, Hardin D, Massion PP. Machine learning models for lung cancer classification using array comparative genomic hybridization. Proc AMIA Symp 2002:7–11.

[33] Daemen A, Gevaert O, Leunen K, Legius E, Vergote I, De Moor B. Supervised classification of array CGH data with HMM-based feature selection. Pac Symp Biocomput 2009:468–.

[34] Jurmeister P, Bockmayr M, Seegerer P, Bockmayr T, Treue D, Montavon G, et al. Machine learning analysis of DNA methylation profiles distinguishes primary lung squamous cell carcinomas from head and neck metastases. Sci Transl Med 2019;11:eaaw8513.

[35] Pohlen T, Hermans A, Mathias M, Leibe B. Full-resolution residual networks for semantic segmentation in street scenes. 30th IEEE Conf Comput Vis Pattern Recognit 2017:3309–18.

[36] Ronneberger O, Fischer P, Brox T. U-Net: convolutional networks for biomedical image segmentation. In: Navab N, Hornegger J, Wells W, Frangi A, editors. Medical image computing and computer-assisted intervention — MICCAI 2015. Cham: Springer; 2015, p.234–141.

[37] Badrinarayanan V, Kendall A, Cipolla R. SegNet: a deep convolutional encoder-decoder architecture for image segmentation. IEEE Trans Pattern Anal Mach Intell 2017;39:2481–95.

[38] Kamnitsas K, Ledig C, Newcombe VFJ, Simpson JP, Kane AD, Menon DK, et al. Efficient multi-scale 3D CNN with fully connected CRF for accurate brain lesion segmentation. Med Image Anal 2017;36:61–78.

[39] Jiang J, Hu YC, Liu CJ, Halpenny D, Hellmann MD, Deasy JO, et al. Multiple resolution residually connected feature streams for automatic lung tumor segmentation from CT images. IEEE Trans Med Imaging 2019;38:134–44.

[40] Wang DD, Zhou W, Yan H, Wong M, Lee V. Personalized prediction of EGFR mutation-induced drug resistance in lung cancer. Sci Rep 2013;3:2855.

[41] Ma L, Wang DD, Zou B, Yan H. An eigen-binding site based method for the analysis of anti-EGFR drug resistance in lung cancer treatment. IEEE/ACM Trans Comput Biol Bioinform 2017;14:1187–94.

[42] Duan B, Zou B, Wang DD, Yan H, Han L. Computational evaluation of EGFR dynamic characteristics in mutation-induced drug resistance prediction. IEEE Int Conf Syst Man Cybern 2015:2299–304.

[43] Zou B, Lee VHF, Yan H. Prediction of sensitivity to gefitinib/erlotinib for egfr mutations in NSCLC based on structural interaction fingerprints and multilinear principal component analysis. BMC Bioinformatics 2018;19:88.

[44] Kureshi N, Abidi SSR, Blouin C. A predictive model for personalized therapeutic interventions in non-small cell lung cancer. IEEE J Biomed Health Inform 2016;20:424–31.

[45] Qureshi R. Personalized drug-response prediction model for lung cancer patients using machine learning. TechRxiv 2020; https://doi.org/10.36227/techrxiv.13273319.v1.

[46] Lecun Y, Bottou L, Bengio Y, Haffner P. Gradient-based learning applied to document recognition. Proc IEEE 1998;86:2278–324.

[47] Szegedy C, Vanhoucke V, Ioffe S, Shlens J, Wojna Z. Rethinking the inception architecture for computer vision. IEEE Conf Comput Vis Pattern Recognit 2016:2818–26.

[48] Kapil A, Meier A, Zuraw A, Steele KE, Rebelatto MC, Schmidt G, et al. Deep semi supervised generative learning for automated tumor proportion scoring on NSCLC tissue needle biopsies. Sci Rep 2018;8:17343.

[49] Tibshirani R, Hastie T, Narasimhan B, Chu G. Diagnosis of multiple cancer types by shrunken centroids of gene expression. Proc Natl Acad Sci U S A 2002;99:6567–72.

[50] Jolliffe IT. A note on the use of principal components in regression. J R Stat Soc C 1982;31:300–3.

[51] Geeleher P, Cox NJ, Huang RS. Clinical drug response can be predicted using baseline gene expression levels and *in vitro* drug sensitivity in cell lines. Genome Biol 2014;15:R47.

[52] Chen YC, Ke WC, Chiu HW. Risk classification of cancer survival using ANN with gene expression data from multiple laboratories. Comput Biol Med 2014;48:1–7.

[53] Liu Y, Yang M, Sun W, Zhang M, Sun J, Wang W, et al. Developing prognostic gene panel of survival time in lung adenocarcinoma patients using machine learning. Transl Cancer Res 2020;9:3860–9.

[54] Yu J, Hu Y, Xu Y, Wang J, Kuang J, Zhang W, et al. LUADpp: an effective prediction model on prognosis of lung adenocarcinomas based on somatic mutational features. BMC Cancer 2019;19:263.

[55] Cho HJ, Lee S, Ji YG, Lee DH. Association of specific gene mutations derived from machine learning with survival in lung adenocarcinoma. PLoS One 2018;13:e0207204.

[56] Yu KH, Berry GJ, Rubin DL, Re C, Altman RB, Snyder M. Association of omics features with histopathology patterns in lung adenocarcinoma. Cell Syst 2017;5:620–7.

[57] Mo Q, Wang S, Seshan VE, Olshen AB, Schultz N, Sander C, et al. Pattern discovery and cancer gene identification in integrated cancer genomic data. Proc Natl Acad Sci U S A 2013;110:4245–50.

[58] Lock EF, Dunson DB. Bayesian consensus clustering. Bioinformatics 2013;29:2610–6.

[59] Nguyen T, Tagett R, Diaz D, Draghici S. A novel approach for data integration and disease subtyping. Genome Res 2017;27:2025–39.

[60] Wang B, Mezlini AM, Demir F, Fiume M, Tu Z, Brudno M, et al. Similarity network fusion for aggregating data types on a genomic scale. Nat Methods 2014;11:333–7.

[61] Ramazzotti D, Lal A, Wang B, Batzoglou S, Sidow A. Multi-omic tumor data reveal diversity of molecular mechanisms that correlate with survival. Nat Commun 2018;9:4453.

[62] Asada K, Kobayashi K, Joutard S, Tubaki M, Takahashi S, Takasawa K, et al. Uncovering prognosis-related genes and pathways by multi-omics analysis in lung cancer. Biomolecules 2020;10:524.

[63] Takahashi S, Asada K, Takasawa K, Shimoyama R, Sakai A, Bolatkan A, et al. Predicting deep learning based multi-omics parallel integration survival subtypes in lung cancer using reverse phase protein array data. Biomolecules 2020;10:1460.

[64] Wiesweg M, Mairinger F, Reis H, Goetz M, Kollmeier J, Misch D, et al. Machine learning reveals a PD-L1-independent prediction of response to immunotherapy of non-small cell lung cancer by gene expression context. Eur J Cancer 2020;140:76–85.

[65] Trebeschi S, Drago SG, Birkbak NJ, Kurilova I, Calin AM, Pizzi AD, et al. Predicting response to cancer immunotherapy using noninvasive radiomic biomarkers. Ann Oncol 2019;30:998–1004.

[66] Coroller TP, Agrawal V, Narayan V, Hou Y, Grossmann P, Lee SW, et al. Radiomic phenotype features predict pathological response in non-small cell lung cancer. Radiother Oncol 2016;119:480–6.

[67] Newman AM, Liu CL, Green MR, Gentles AJ, Feng W, Xu Y, et al. Robust enumeration of cell subsets from tissue expression profiles. Nat Methods 2015;12:453–7.

[68] Tosolini M, Pont F, Poupot M, Vergez F, Nicolau-Travers M-L, Vermijlen D, et al. Assessment of tumor-infiltrating TCRVγ9Vδ2 γδ lymphocyte abundance by deconvolution of human cancers microarrays. Oncoimmunology 2017;6:e1284723.

[69] Sun R, Limkin EJ, Vakalopoulou M, Dercle L, Champiat S, Han SR, et al. A radiomics approach to assess tumour-infiltrating CD8 cells and response to anti-PD-1 or anti-PD-L1 immunotherapy: an imaging biomarker, retrospective multicohort study. Lancet Oncol 2018;19:1180–91.

[70] Zhao T, Hou L, Nguyen V, Gao Y, Samaras D, Kurc T, et al. Using machine methods to score tumor-infiltrating lymphocytes in lung cancer. USCAP Annual Meeting 2017;97:403A.

[71] Saltz J, Gupta R, Hou L, Kurc T, Singh P, Nguyen V, et al. Spatial organization and molecular correlation of tumor-infiltrating lymphocytes using deep learning on pathology images. Cell Rep 2018;23:181–93.

[72] Lundegaard C, Lamberth K, Harndahl M, Buus S, Lund O, Nielsen M. NetMHC-3.0: accurate web accessible predictions of human, mouse and monkey MHC class I affinities for peptides of length 8–11. Nucleic Acids Res 2008;36:W509–12.

[73] O’Donnell TJ, Rubinsteyn A, Bonsack M, Riemer AB, Laserson U, Hammerbacher J. MHCflurry: open-source class I MHC binding affinity prediction. Cell Syst 2018;7:129–32.

[74] Nielsen M, Lundegaard C, Blicher T, Lamberth K, Harndahl M, Justesen S, et al. NetMHCpan, a method for quantitative predictions of peptide binding to any HLA-A and -B locus protein of known sequence. Plos One 2007;2:e796.

[75] Bulik-Sullivan B, Busby J, Palmer CD, Davis MJ, Murphy T, Clark A, et al. Deep learning using tumor HLA peptide mass spectrometry datasets improves neoantigen identification. Nat Biotechnol 2019;37:55–63.
